# Self-perceived competencies in the diagnosis and treatment of mental health disorders among general practitioners in Lima, Peru

**DOI:** 10.1186/s12909-019-1900-8

**Published:** 2019-12-16

**Authors:** Jessica Hanae Zafra-Tanaka, Kevin Pacheco-Barrios, Fiorella Inga-Berrospi, Alvaro Taype-Rondan

**Affiliations:** 1 CRONICAS Center of Excellence for Chronic Diseases, Universidad Peruana Cayetano Heredia, Lima, Peru; 2 SYNAPSIS Mental Health and Neurology, Non-Profit organization, Lima, Peru; 3 Unidad de Investigación para la Generación y Síntesis de Evidencias en Salud, Universidad San Ignacio de Loyola, Lima, Peru; 4 Universidad Continental, Lima, Peru

**Keywords:** Mental health, Psychiatry, Self-perceived competence, Skills, Undergraduate education

## Abstract

**Aim:**

To assess the self-perceived competencies in diagnosing and treating patients with mental health disorders, among recently graduated general practitioners (GPs) from Lima, Peru.

**Methods:**

A cross-sectional study was performed in April 2017 at a General Practitioner’s meeting held for those who were going to perform the social service, by the Peruvian College of Physicians in Lima. Attendees were invited to answer a questionnaire that evaluated their self-perception of competence in diagnosing and treating four different mental health disorders; major depression, anxiety disorder, alcohol dependence, and schizophrenia.

**Results:**

Out of 434 evaluated GPs, the following percentages were self-perceived as competent in their adequate diagnosis of depression (70.5%), anxiety (73.3%), alcohol dependence (67.6%), and schizophrenia (62.0%). Concerning pharmacological treatment, these percentages were 46.6, 47.5, 39.0 and 37.6%, respectively. Referring to all the studied mental disorders, 41.6% of participants self-perceived competence in providing an adequate diagnosis, 36.1% in providing non-pharmacological treatment, and 20.1% in providing pharmacological treatment.

**Conclusion:**

The rate of adequate self-perceived competences was higher for diagnosis than for treatment of patients with mental health disorders. These results highlight the importance of designing and implementing interventions to improve medical education so as to develop the skills necessary to confront mental health disorders.

## Background

The burden of mental health disorders continues to grow over time, with 22.7% of people worldwide now living with a mental health disability [1]. Regrettably, the number of specialized mental health professionals is insufficient to confront this problem, this is predominantly tangible in developing countries where the ratio of physicians to patients is much more disparate [2, 3]. Thus, it is necessary for primary health care personnel such as general practitioners (GPs), psychologists, nurses, and technical staff to be able to diagnose and treat mental health disorders.

In Peru, 6 out of 24 regions have no psychiatrist in the public health care system, and in the regions that do have any psychiatrist, they are generally restricted to large cities [3] and urban areas, leaving rural areas without access to mental health care. GPs are usually in charge of diagnosing and treating patients with mental health disorders in primary care [4, 5]. Many GPs working in primary care are currently performing the Urban Rural Marginal Internship Program (called SERUMS for its acronym in Spanish), which is a social service mandatory for those who want to work for the state or carry out medical residency and studies in Perú [6, 7]. The SERUMS program takes place in underserved rural areas where there are no specialists, so the GPs working on their SERUMS are in charge of managing mental health disorders. This makes it essential for recently graduated GPs to receive suitable mental health care training and education in medical schools.

Worldwide, most studies on mental health capacities among GPs have assessed their knowledge [8–10], while few have assessed their competencies on the diagnosis or treatment of mental disorders [11–16], none of them in Peru. These studies have found low self-perceived competencies regarding this issue. Even though these studies have evaluated heterogeneous groups of GPs, none of them have assessed recently graduated GPs, particularly relevant in Peru.

This scarcity of information makes it difficult to develop and implement educational interventions to improve the diagnosis and treatment skills of mental health disorders among GPs in Peru. For this reason, the objectives of the present study were: 1) to assess the self-perceived competencies in the diagnosis and treatment of mental health disorders among recently graduated GPs from Lima, Peru, 2) to evaluate the association between self-perceived competencies and the university were the GP studied, and 3) to explore aspects to be improved in undergraduate psychiatry courses. This will provide decision makers and professors a general picture of the needs GPs perceive and help identify which universities are the ones that need to improve more.

## Method

### Type of study and population

A cross-sectional study was performed in which self-administered surveys were presented to GPs attending the “Sixth National SERUMS Convention” (“VI Convención Nacional SERUMS”), a convention organized by The Young Physicians Committee (“Comité Médico Joven”) of the Peruvian College of Physicians in April 2017. This convention aimed to provide general information and insights of the SERUMS program to the GPs who planned to form part of the program in 2017.

All GPs who registered their attendance at the convention and agreed to participate in the study were included. For data analysis, those who did not complete their undergraduate studies in Lima, or who had not recently graduated (in the last year), or did not completely fill out the variables of interest in the survey, were excluded.

### Study procedures

Trained personnel handed the study survey to all the participants at the time they entered the convention. Each GP was given an oral explanation of the study along with a summary of the study and the survey. The survey was anonymously completed before the start of the convention and during the recess. The researchers were present to response any questions the participants might have regarding the survey and to collect the questionnaires once they were finished.

The data was double-entered by different researchers in an independent fashion. A researcher who did not participate in this process cross-checked the double-entered data, and in cases of discrepancies, the surveys were re-evaluated to correct any mistakes.

### Survey

An ad hoc survey was created for the study, which included the following four sections: 1) sociodemographic data, 2) self-perception of competencies in mental health, 3) self-perception of competencies in general medicine, and 4) self-perception of competencies in obstetric care. For this study, we used data from the first two sections.

The mental health section assessed self-perceived competencies in the four mental health disorders with the highest worldwide burden in terms of years lived with a disability, as reported by the World Health Organization [1]: major depression, anxiety disorder, alcohol dependency, and schizophrenia. There was also a subsection included with five questions which aimed to assess areas of potential improvement in undergraduate psychiatry courses. To assure the content validity and ensure that the questions proposed were adequate and understandable, our survey was reviewed by psychiatrists and by recently graduated GPs who did not take part in the study.

### Study variables

#### Perception of competencies in mental health

Four mental health disorders were studied: major depression, anxiety disorder, alcohol dependency, and schizophrenia. For the first three disorders, the following competencies were assessed: diagnosis, non-pharmacological treatment (e.g. psychoeducation), and pharmacological treatment; while only diagnosis and pharmacological treatment were assessed for schizophrenia.

Each competency was assessed using the following statement: “I have adequate competencies for ...” and five possible responses (strongly disagree, disagree, neutral, agree, and strongly agree). We considered self-perception of adequate competence when the GP answered “agree” or “strongly agree” on all the statements for each variable assessed (diagnosis, pharmacological treatment, and non-pharmacological treatment). This was a dichotomous variable with possible answers “yes” or “no”.

#### Aspects to be improved in undergraduate psychiatry courses

We also asked about aspects to be improved in undergraduate psychiatry courses using the following question: “The psychiatry course in my faculty should have ...” with five aspects (more critical reading of scientific articles, more theoretical classes, more hours in the outpatient clinic, more contact with patients, and more teaching about patient treatment). For each aspect, five possible answers were proposed (strongly disagree, disagree, neutral, agree, and strongly agree).

#### Other variables

Other variables evaluated were age, sex, year in which the GP completed their internship, and the university where the GP studied medicine. Age was categorized in tertiles (22 to 25, 26, and 27 to 42 years) to facilitate the analysis and interpretation of data.

### Statistical analysis

For the descriptive analysis of categorical variables, absolute and relative frequencies were used. For the sociodemographic quantitative variables, means and their respective standard deviations were reported.

In addition, we assessed the association between the university where the GP studied and three outcomes: self-perception of adequate competence in the diagnosis, non-pharmacological, and pharmacological treatment of all mental health disorders. For this, prevalence ratios (PR) and their 95% confidence intervals (95% CI) were calculated using Poisson regression with robust variance. We also performed power analysis to evaluate the probability of making type 2 error. In order to estimate the power of each of our associations, we estimated power for a PR of 1.5, a level of significance of 95%, and a proportion of the comparison group of 40% for diagnostic and for non-pharmacological management, and of 20% for pharmacological management; according to the rate of these outcomes.

Also, we performed a Kruskal Wallis test to evaluate the differences between the universities when analyzing our primary outcomes as continuous variables. In order to so, we coded the five possible answers as follows: strongly disagree = 1, disagree = 2, neutral = 3, agree = 4, and strongly agree = 5. Using these values, we estimated a mean score for each outcome (diagnostics, pharmacological treatment, and non-pharmacological treatment) by adding all the values obtained for each medical condition (major depression, anxiety disorder, alcohol dependency, and schizophrenia), and dividing it by the number of conditions. We used the Dunn test as a post hoc analysis to perform pairwise comparison.

Given the small proportion of missing data (0.9 to 3% for the questions that aimed to assess competence), we decided that, when data was not available for certain participant, we would not include it in the analysis. That said, we did not perform any type of imputation when data was not available for certain participant, we would not include it in the analysis. That said, we did not perform any type of imputation. Analyses were performed using Stata software v14.0.

## Results

Of the 688 GPs who were given the survey during enrollment at the convention, 520 (75.6%) responded. Of these, 66 were excluded because they had undergone undergraduate studies outside Lima, and 20 that were not recently graduated GPs (i.e., those who finished medical school before 2016), leaving 434 for analysis. This represents 33.2% of all GPs recently graduated from universities in Lima according to the Peruvian College of physicians. Among the evaluated GPs, the mean age was 26.01 ± 2.8 years, and 246 (56.7%) were female (Table 1).

**Table 1:** Characteristics of evaluated recently graduated general practitioners from Lima, Peru (*N* = 434)

Characteristics^a^	N (%)
Age (years old) (*n* = 422)	
22 to 25	222 (52.6)
26	73 (17.3)
27 to 42	127 (30.1)
Gender (*n* = 434)	
Males	188 (43.3)
Females	246 (56.7)
University (*n* = 434)	
USMP	119 (27.4)
UNMSM	69 (15.9)
URP	64 (14.8)
UCSur	56 (12.9)
UPSJB	47 (10.8)
UNFV	42 (9.7)
UPCH	24 (5.5)
UPC	13 (3.0)

^a^Totals do not add up to 434 because of missing data

*USMP* Universidad San Martín de Porres, *UNMSM* Universidad Nacional Mayor de San Marcos, *URP* Universidad Ricardo Palma, *UCSur* Universidad Científica del Sur, *UPSJB* Universidad Privada San Juan Bautista, *UNFV* Universidad Nacional Federico Villareal, *UPCH* Universidad Peruana Cayetano Heredia, *UPC* Universidad Peruana de Ciencias Aplicadas

**Fig. 1:**
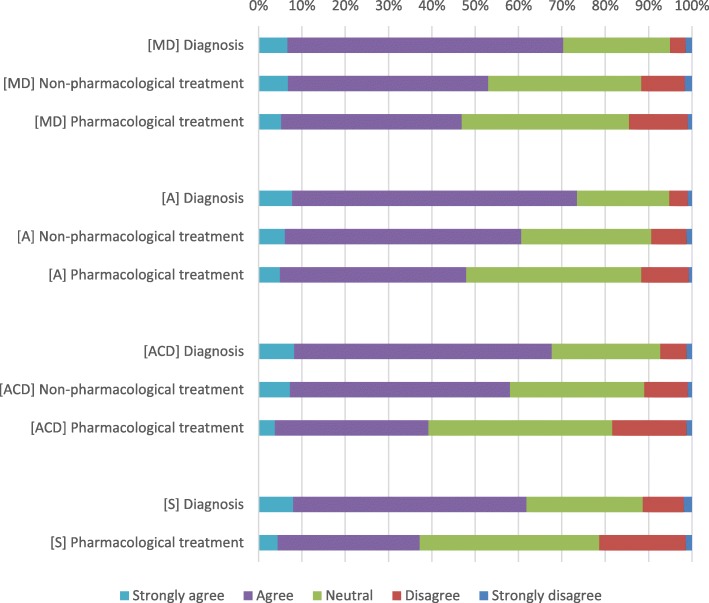
Self-perceived competencies among recently graduated general practitioners in Lima, Peru. Answers to the question “I have adequate competencies to ...” are shown in the figure. MD: major depression disorder, A: Anxiety, ACD: Alcohol dependency disorder, S: schizophrenia

With respect to clinical depression, 296 (70.3%) GPs perceived themselves to be adequately competent to perform an adequate diagnosis, 227 (53.0%) to provide non-pharmacological treatment, and 198 (46.9%) to provide pharmacological treatment. With respect to anxiety disorder, 311 (73.5%) perceived themselves to be sufficiently competent to perform an adequate diagnosis, 259 (60.7%) to provide non-pharmacological treatment, and 205 (47.9%) to provide pharmacological treatment. Regarding alcohol dependency disorder, 289 (67.7%) perceived themselves to be sufficiently competent to perform an adequate diagnosis, 249 (58.0%) to provide non-pharmacological treatment, and 167 (39.2%) to provide pharmacological treatment. With respect to schizophrenia, 263 (61.9%) perceived themselves to be sufficiently competent to perform diagnosis, and 160 (37.2%) to provide pharmacological treatment (Fig. 1). Missing data from the questions that aimed to assess competence ranged from 0.9 to 3%.

From the evaluated GPs, 170 (41.6%) perceived themselves to be adequately competent to perform the diagnosis, 153 (36.1%) to provide non-pharmacological treatment, and 84 (20.1%) to provide the pharmacological treatment for the four assessed mental health disorders. These proportions varied across universities (Table 2). Thus, in the case of diagnosis, percentages were higher among those who completed their undergraduate education at Universidad Nacional Mayor de San Marcos (UNMSM) (PR: 1.88, 95% CI: 1.52–2.34) or Universidad Peruana Cayetano Heredia (UPCH) (PR: 1.62, 95% CI: 1.18–2.24). While in the case of pharmacological treatment, percentages were higher among those who completed their undergraduate education at UNMSM (PR: 1.61, 95% CI: 1.05–2.48) or UPCH (PR: 1.98, 95% CI: 1.13–3.44) (Table 2).

**Table 2:** University and percentage of general practitioners self-perceived as adequately competent to perform the diagnosis, non-pharmacological, and pharmacological treatment of four mental disorders (*N* = 434)

University	Diagnosis		Non-pharmacological treatment		Pharmacological treatment	
	n/N (%)	PR (95% CI)^a^	n/N (%)	PR (95% CI)^a^	n/N (%)	PR (95% CI)^a^
USMP	33/113 (29.2)	**0.63 (0.46–0.86)**	39/118 (33.1)	0.89 (0.66–1.19)	16/118 (13.6)	**0.60 (0.36–0.99)**
UNMSM	44/64 (68.8)	**1.88 (1.52–2.34)**	28/68 (41.2)	1.17 (0.85–1.61)	20/68 (29.4)	**1.61 (1.05–2.48)**
URP	22/58 (37.9)	0.90 (0.63–1.28)	22/63 (34.9)	0.96 (0.67–1.39)	9/57 (15.8)	0.76 (0.40–1.44)
UCSur	16/54 (29.6)	0.68 (0.45–1.05)	19/55 (34.5)	0.95 (0.65–1.40)	9/55 (16.4)	0.79 (0.42–1.49)
UPSJB	22/47 (46.8)	1.14 (0.82–1.59)	15/44 (34.1)	0.94 (0.61–1.45)	11/45 (24.4)	1.25 (0.72–2.18)
UNFV	14/38 (36.8)	0.88 (0.57–1.35)	13/40 (32.5)	0.89 (0.56–1.42)	7/39 (17.9)	0.89 (0.44–1.79)
UPCH	15/23 (65.2)	**1.62 (1.18–2.24)**	12/23 (52.2)	1.48 (0.98–2.24)	9/24 (37.5)	**1.98 (1.13–3.44)**
UPC	4/12 (33.3)	0.80 (0.35–1.79)	5/13 (38.5)	1.07 (0.53–2.15)	3/13 (23.1)	1.16 (0.42–3.18)

^a^Each university group was compared with the rest of universities. Bold values denote statistical significance

*USMP* Universidad San Martín de Porres, *UNMSM* Universidad Nacional Mayor de San Marcos, *URP* Universidad Ricardo Palma, *UCSur* Universidad Científica del Sur, *UPSJB* Universidad Privada San Juan Bautista, *UNFV* Universidad Nacional Federico Villareal, *UPCH* Universidad Peruana Cayetano Heredia, *UPC* Universidad Peruana de Ciencias Aplicadas

We also performed power analysis for all of the above-mentioned comparisons, and found that, in the case of diagnostic and non-pharmacological treatment our power was less than 80% to find a PR of 1.5 for the following universities: UCSur, UPSJB, UNFV, UPCH, and UPC. In the case of pharmacological treatment, we found that all of the comparisons had a power of less than 80% to find a PR of 1.5. (Additional file 2).

When analyzing the primary outcomes as continuous variables using the Kruskal Wallis test, we found an association between the university and the perception of being competent to perform diagnosis using Kruskal Wallis test (H(7) = 41.63, *p* value ≤0.001). We found differences between UNMSM and USMP (*p* < 0.001), URP (*p* = 0.005), UCSur (*p* = 0.002), UNFV (*p* = 0.020); and between UPCH and USMP (*p* < 0.001), URP (*p* = 0.016), UCSur (*p* = 0.008), and UNFV (*p* = 0.033).

We also found an association between the university and the perception of being competent to perform pharmacological treatment (H(7) = 22.79, *p* value = 0.002). We found differences between UPCH and USMP (*p* = 0.006), URP (*p* = 0.004), and UCSur (*p* = 0.004).

On the other hand, we found no association between the university and the perception of being competent to provide non-pharmacological treatment (H(7) = 11.54, *p* value = 0.117).

**Fig. 2:**
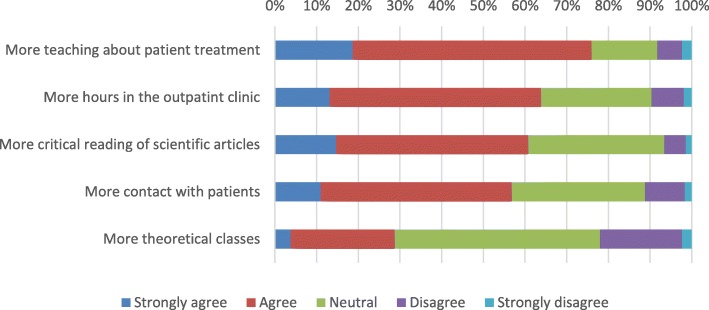
Recommendations to improve the undergraduate courseware in psychiatry among recently graduated general practitioners in Lima, Peru

From the evaluated GPs, 75.9% recommended that coursework in psychiatry should have further training on patient treatment, 63.9% responded it should have more hours in the outpatient clinic, 60.8% responded that it should have more critical reading of scientific articles, 56.9% responded that it should have more contact with patients, and 28.7% that it should have more theoretical classes (Fig. 2).

## Discussion

We found that round 7 of 10 of the GPs surveyed self-perceived as competent in the diagnosis and around 5 in 10 self-perceived competent in the treatment of the studied disorders. In general, around four in ten GPs self-perceived themselves as competent in performing diagnosis and in providing non-pharmacological treatment for all the studied mental health disorders, while two in ten GPs self-perceived themselves as competent to provide pharmacological treatment for all the studied mental health disorders. The self-perception of competence in performing diagnosis and in providing pharmacological treatment was greater among those who finished their undergraduate studies at UPCH and UNMSM than those who did not.

In Peru, the percentage of GPs with self-perception of adequate competencies for the diagnosis and treatment of mental health disorders was low. Previous studies found similar results: in Brazil, 10/24 (41.7%) GPs detected that patients had mental health disorders when assessing a patient’s mental status blinded to the results of the General Health Questionnaire (GHQ-12) [8]. In Peru, a study in 52 primary health care professionals which included 6 GPs and 19 psychiatrists, reported that 16% of participants reported “a great deal” of training on drugs and alcohol during undergraduate courses [16]. In Nepal, a study of 29 primary health care professionals (12 GPs) reported that clinical rotations in psychiatry during their undergraduate studies ranged from 0 to 15 days and were considered inadequate [13]. In Australia, a study in 256 GPs found that 19.4% felt very confident making the diagnosis of mental health disorders, and 94.7% felt very or somewhat confident in prescribing antidepressants, 83.2% prescribing sedatives, and 50% prescribing antipsychotics [14]. Compared to Australian physicians, Peruvian recently graduated physicians felt less confident in providing pharmacological treatment for the above-mentioned mental health disorders.

We found that those who studied in UNMSM and UPCH had a greater percentage of GPs that perceived themselves as sufficiently competent to perform an adequate diagnosis and treatment of the four mental health disorders studied. It should be noted that UNMSM and UPCH are the universities that usually occupy the top positions in the international university rankings in human medicine in Peru [17, 18] and in the Peruvian National Medical Board Exam [19]. The differences found may be due to differences in the curricular structure of undergraduate mental health courses [20]. For instance, the psychiatry course in the UNMSM has 68 h of theoretical work and 136 h of practice, UPCH has 50 h of theoretical work and 100 h of practice, whereas other universities had fewer hours [21–23]. However, many other factors may be involved in these differences also.

Given that only four in ten GPs self-perceived as competent in providing non-pharmacological treatment for all the mental health disorders studied, and that there was no difference between competencies versus universities, we believe that these aspects of medical training are not thoroughly taught during medical school at all the universities included in the study.

It should be noted that the population of this study were GPs who studied at universities in Lima, the Peruvian city with the most psychiatrists [24]. However, in other cities in Peru the number of psychiatrists is very low, which may be affecting mental health education.

Our results have important implications for Peruvian medical schools, since according to Peruvian accreditation criteria for medical schools, GPs should be capable of diagnosing and treating mental health disorders [25]. For this reason, it is necessary to assess the characteristics of undergraduate curricula and mental health courses [26, 27]. These are functions attributed by law to the National Superintendence of Higher University Education (SUNEDU), a public body associated with the Ministry of Education [28].

Some authors have highlighted that undergraduate training in mental health should achieve the following competencies: adequate diagnosis and doctor–patient relationships, basic treatment principles, recognition of behaviors and/or emotions that interfere with care, and medical care based on a multidisciplinary team [20, 27]. To do so, it would be important to consider the suggestions made by the respondents, such as enhancing the time for discussion of scientific articles and a clerkship in first level of care centers. Finally, due to the fact that most of recently graduated GPs in Peru perform their social service in rural settings, it is necessary to reinforce a systematic integration of these competencies with rural cultural features in mental health education [29].

Our results also have implications for the health care system, since health personnel with adequate competencies in mental health are needed in primary care facilities. Thus, GPs with these concerns prior to the SERUMS program could improve their ability and predisposition to manage patients with mental health disorders [11] and increase the number of referrals and the number of people identified and treated for mental health disorders [30]. For this to take place, courses that allow massive group training of health care personnel, including free virtual courses that address the treatment of mental health disorders [22], and the World Health Organization-driven Mental Health Gap Action Program (mhGAP) [2] could be used.

Some limitations must be considered: 1) we have assessed self-perceived competencies, which would not necessarily reflect the actual competencies of these GPs, as suggested previously for competencies not related to mental health [31]. However, we consider that the assessment of self-perceived competencies (the idea that each participant has regarding their own competence to perform a task) is important as it is closely related to motivation and outlook, which can directly influence their decisions about performing a task or not [32, 33]. 2) Our sample represented 23.0% of all GPs graduated in 2016 in Lima, and included different proportions of GPs from each university (see Additional file 1). Moreover, we were not able to compare characteristics of all graduated physicians in 2016 and our sample. However, since the vast majority of recently graduated GPs are applying for SERUMS program, it is possible that those who did not attend the conference in which our survey was performed were not informed about it or because they had other commitments, this not likely being a large bias in our results. 3) when analyzing the competencies by university were the GP finished their undergraduate studies, we have a small number of GPs from some universities such as UPC or UPCH, which translates into imprecision of the effect and low power. This prevents us from concluding that there is a lack of association between these universities and the outcomes.

Nonetheless, this is the first study to assess competencies in the valuation of mental health disorders in recently graduated GPs in Peru, who are the ones who provide mental health services in primary care facilities. Moreover, our results indicate a deficiency in Peruvian medical education that calls for intervention in the design and implementation of corresponding education.

## Conclusion

Among recently graduated GPs, the rate of self-perceived adequate competencies for diagnosis and management of mental health disorders was low. These proportions differed across universities. These results highlight the importance of assessing and implementing interventions in undergraduate medical education in mental health care in Peru.

## Supplementary information

**Additional file 1.** Recently graduated general practitioners included in the study compared to all recently graduated general practitioners from universities of Lima, Peru.

**Additional file 2.** Proportion of general practitioners self-perceived as adequately competent to perform the diagnosis, non-pharmacological treatment, and pharmacological treatment of four mental disorders; and statistical power for the comparisons.

## Data Availability

The datasets used and/or analyzed during the current study are available on reasonable request from the corresponding author.

## Abbreviations

95% CI: 95% confidence intervals
GHQ-12: General Health Questionnaire
GP: General practitioners
mhGAP: Mental Health Gap Action Program
PR: Prevalence ratio
SERUMS: Urban Rural Marginal Internship Program
SUNEDU: National Superintendence of Higher University Education
UCSur: Universidad Científica del Sur
UNFV: Universidad Nacional Federico Villareal
UNMSM: Universidad Nacional Mayor de San Marcos
UPC: Universidad Peruana de Ciencias Aplicadas
UPCH: Universidad Peruana Cayetano Heredia
UPSJB: Universidad Privada San Juan Bautista
URP: Universidad Ricardo Palma
USMP: Universidad San Martín de Porres
