# Supplementary material for: Self-perceived competencies in the diagnosis and treatment of mental health disorders among general practitioners in Lima, Peru
Source: BMC Med Educ. 2019 Dec 16;19:464. doi: 10.1186/s12909-019-1900-8 (PMC6916154; doi:10.1186/s12909-019-1900-8)
Supplement: Supplementary file 1 — Additional file 1. Recently graduated general practitioners included in the study compared to all recently graduated general practitioners from universities of Lima, Peru. [file 12909_2019_1900_MOESM1_ESM.docx]

## Supplementary material - 1. Recently graduated general practitioners included in the study versus all recently graduated general practitioners from universities of Lima, Peru

|  | General practitioners graduated from med-school in 2016* | General practitioners included in the study |
| --- | --- | --- |
| USMP | 342 | 119 (46.9%) |
| UNMSM | 147 | 69 (34.8%) |
| URP | 177 | 64 (36.2%) |
| UCSur | 150 | 56 (37.3%) |
| UPSJB | 250 | 47 (18.8%) |
| UNFV | 105 | 42 (40.0%) |
| UPCH | 120 | 24 (20.0%) |
| UPC | 77 | 13 (16.9%) |
| Total | 1368 | 315 (23.0%) |
| Recently graduated: those who finished their studies on 2016.  USMP: Universidad San Martín de Porres, UNMSM: Universidad Nacional Mayor de San Marcos, URP: Universidad Ricardo Palma, UCSur: Universidad Científica del Sur, UPSJB: Universidad Privada San Juan Bautista, UNFV: Universidad Nacional Federico Villareal, UPCH: Universidad Peruana Cayetano Heredia, UPC: Universidad Peruana de Ciencias Aplicadas | | |
| *Source: “Colegio Medico Joven” of the Peruvian College of physicians. | | |
