# Supplementary material for: Self-perceived competencies in the diagnosis and treatment of mental health disorders among general practitioners in Lima, Peru
Source: BMC Med Educ. 2019 Dec 16;19:464. doi: 10.1186/s12909-019-1900-8 (PMC6916154; doi:10.1186/s12909-019-1900-8)
Supplement: Supplementary file 2 — Additional file 2. Proportion of general practitioners self-perceived as adequately competent to perform the diagnosis, non-pharmacological treatment, and pharmacological treatment of four mental disorders; and statistical power for the comparisons. [file 12909_2019_1900_MOESM2_ESM.docx]

## Supplementary material 2. University and proportion of general practitioners self-perceived as adequately competent top perform the diagnosis, non-pharmacological, and pharmacological treatment of four mental disorders

| **University** | Diagnosis | | Non-pharmacological treatment | | Pharmacological treatment | |
| --- | --- | --- | --- | --- | --- | --- |
|  | PR (95% CI)* | Power** | PR (95% CI)* | Power** | PR (95% CI)* | Power** |
| USMP | 0.63 (0.46 - 0.86) | 0.9554 | 0.89 (0.66 - 1.19) | 0.9554 | 0.60 (0.36 - 0.99) | **0.5739** |
| UNMSM | **1.88 (1.52 - 2.34)** | 0.8457 | 1.17 (0.85 - 1.61) | 0.8457 | **1.61 (1.05 - 2.48)** | **0.4373** |
| URP | 0.90 (0.63 - 1.28) | 0.8161 | 0.96 (0.67 - 1.39) | 0.8161 | 0.76 (0.40 - 1.44) | **0.4138** |
| UCSur | 0.68 (0.45 - 1.05) | **0.7929** | 0.95 (0.65 - 1.40) | **0.7929** | 0.79 (0.42 - 1.49) | **0.3972** |
| UPSJB | 1.14 (0.82 - 1.59) | **0.7443** | 0.94 (0.61 - 1.45) | **0.7443** | 1.25 (0.72 - 2.18) | **0.3662** |
| UNFV | 0.88 (0.57 - 1.35) | **0.6639** | 0.89 (0.56 - 1.42) | **0.6639** | 0.89 (0.44 - 1.79) | **0.3225** |
| UPCH | **1.62 (1.18 - 2.24)** | **0.4735** | 1.48 (0.98 - 2.24) | **0.4735** | **1.98 (1.13 - 3.44)** | **0.2396** |
| UPC | 0.80 (0.35 - 1.79) | **0.2843** | 1.07 (0.53 - 2.15) | **0.2843** | 1.16 (0.42 - 3.18) | **0.1705** |
| * Each university group was compared with the rest of universities.  USMP: Universidad San Martín de Porres, UNMSM: Universidad Nacional Mayor de San Marcos, URP: Universidad Ricardo Palma, UCSur: Universidad Científica del Sur, UPSJB: Universidad Privada San Juan Bautista, UNFV: Universidad Nacional Federico Villareal, UPCH: Universidad Peruana Cayetano Heredia, UPC: Universidad Peruana de Ciencias Aplicadas  **Estimated power for a PR of 1.5, a level of significance of 95%, and a proportion of the comparison group of 40% for diagnostic and for non-pharmacological management, and of 20% for pharmacological management | | | | | | |
